# Real-world evidence of remdesivir in formerly hospitalized COVID-19 patients: patient-reported and functional outcomes

**DOI:** 10.1186/s12879-024-10398-w

**Published:** 2025-01-09

**Authors:** Dorottya Fésü, Enikő Bárczi, Balázs Csoma, Lőrinc Polivka, Márton Boga, Gábor Horváth, János Tamás Varga, Szilvia Sebők, Veronika Müller

**Affiliations:** 1 Department of Pulmonology, Semmelweis University, Budapest, Hungary; 2 Heart and Vascular Centre, Semmelweis University, Budapest, Hungary; 3 Department of Pharmacy Administration, University Pharmacy, Semmelweis University, Budapest, Hungary

**Keywords:** Post-infectious disorders, Post-acute COVID-19 syndrome, Symptom burden, Antiviral agents, Remdesivir, Quality of life

## Abstract

**Background:**

Post-COVID condition (PCC) is characterized by persisting symptoms after the resolution of acute COVID-19. Remdesivir (RDV), a broad-spectrum antiviral drug, has been widely used in patients hospitalized with COVID-19 requiring oxygen therapy. We aimed to evaluate the effects of RDV on PCC by assessing patient-reported and functional outcomes.

**Methods:**

We used the data from a single-center registry, including formerly hospitalized post-COVID patients (*N* = 293). Propensity score matching (PSM) was used (16 criteria, 1:1 ratio) to obtain two comparable groups: those who received standard-of-care (SOC, *N* = 94) and those treated with RDV in addition to SOC (SOC + RDV, *N* = 94). Primary outcomes were asymptomatic status and at least 50% symptom score reduction at post-COVID follow-up. Secondary outcomes included results of pulmonary function (PF) tests, 6-minute walk test (6MWT), and quality-of-life (QoL) questionnaires.

**Results:**

After PSM, baseline patient characteristics showed no significant differences between the two groups. Most patients were still symptomatic (60% vs. 66%). In the SOC + RDV group, the use of oxygen supplementation (94 vs. 80%, *p* = 0.005) and steroids (97 vs. 88%, *p* = 0.027) during infection were higher, while patients presented at their post-COVID visits earlier (median 68 vs. 97 days, *p* = 0.003). Complete or at least 50% symptom resolution were reported at a significantly earlier stage after infection in the SOC + RDV group compared to the SOC group (multivariable-adjusted HR = 2.28, 95% CI = 1.33–3.92, *p* = 0.003; and HR = 2.08, 95% CI = 1.43–3.02, *p* < 0.001; respectively). In the SOC + RDV group, fewer patients experienced sleep disturbances at PCC, and sleep-related questionnaires (Pittsburg Sleep Quality Index, PSQI) results showed significantly better sleep quality (14 vs. 27% and 5.9 vs. 7.7 points, respectively). There were no notable differences in results of PF tests, 6MWT, and other QoL questionnaires.

**Conclusion:**

In this propensity score matched cohort, the use of RDV was associated with earlier patient reported symptom resolution during the PCC period, while there were no notable differences in functional outcomes. Our results indicate a possible beneficial effect of RDV in terms of faster symptom resolution after COVID19 infection.

**Graphical abstract:**

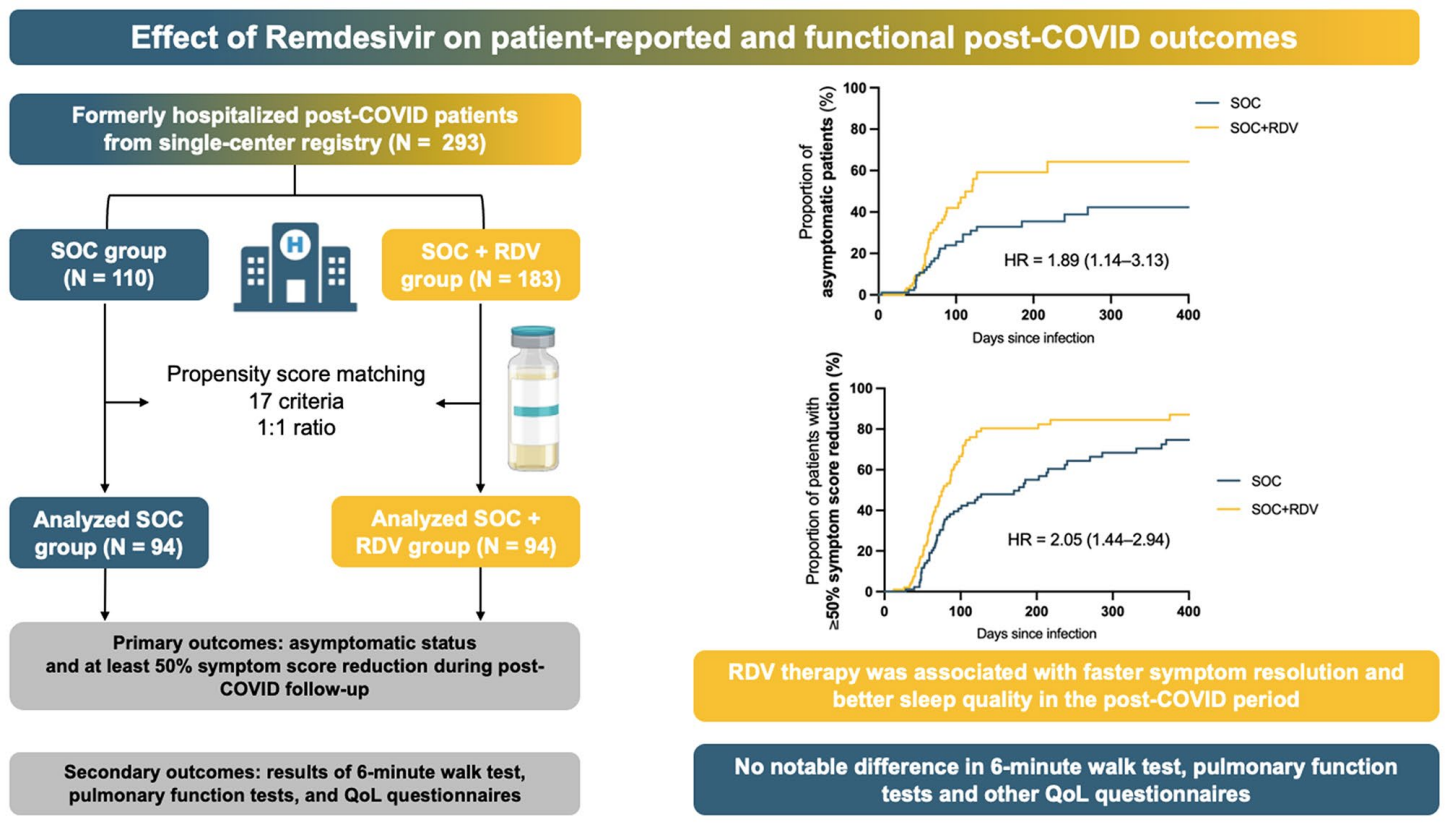

**Supplementary Information:**

The online version contains supplementary material available at 10.1186/s12879-024-10398-w.

## Introduction

The outbreaks of SARS-CoV2 (severe acute respiratory syndrome coronavirus 2) pandemic resulted in many hospitalized patients globally. Especially pre-vaccination waves – original Wuhan and Alfa variants of concern (VOC) – and the Delta VOC were associated with a high burden of pneumonia needing hospital care and often requiring different forms of oxygen supplementation [1–4].

Post-COVID condition (PCC) is defined as a long-term condition with several manifestations of multisystem disease, including different persisting symptoms, following mild to severe COVID-19 [5–8]. The most common complaints are fatigue and pulmonary symptoms, including dyspnea, cough, chest pain, decreased exercise capacity, and sleep disturbances [9–11].

The antiviral drug remdesivir (RDV) was approved by the Food and Drug Administration (FDA) [12] and the European Medicines Agency (EMA) [13] for the treatment of COVID-19 and has been widely used as a treatment option for COVID-19. During the first waves of the SARS-CoV2 pandemic, RDV was used in patients needing low to high-flow oxygen therapy or non-invasive ventilation [14–16]. However, there were conflicting data on the short-term benefits of RDV therapy in patients requiring invasive ventilation [17–21]. Therefore, during the initial waves of the pandemic, RDV was not administered to patients in need of invasive ventilation, with high liver enzymes or underlying kidney disease according to the available EMA and FDA summary of product characteristics (SmPC) at that time [12, 16, 22]. However, the label was extended according to subsequent studies that also confirmed its use in the case of kidney disease [23, 24]. The long-term post-infection effects of RDV are not well-known yet, and data is scarce particularly on PCC symptom burden and quality of life (QoL) after infection [25, 26]. We aimed to fill this knowledge gap by evaluating the effect of RDV in post-COVID patients with special emphasis on the resolution of symptom burden.

## Materials and methods

Study population.

**Fig. 1:**
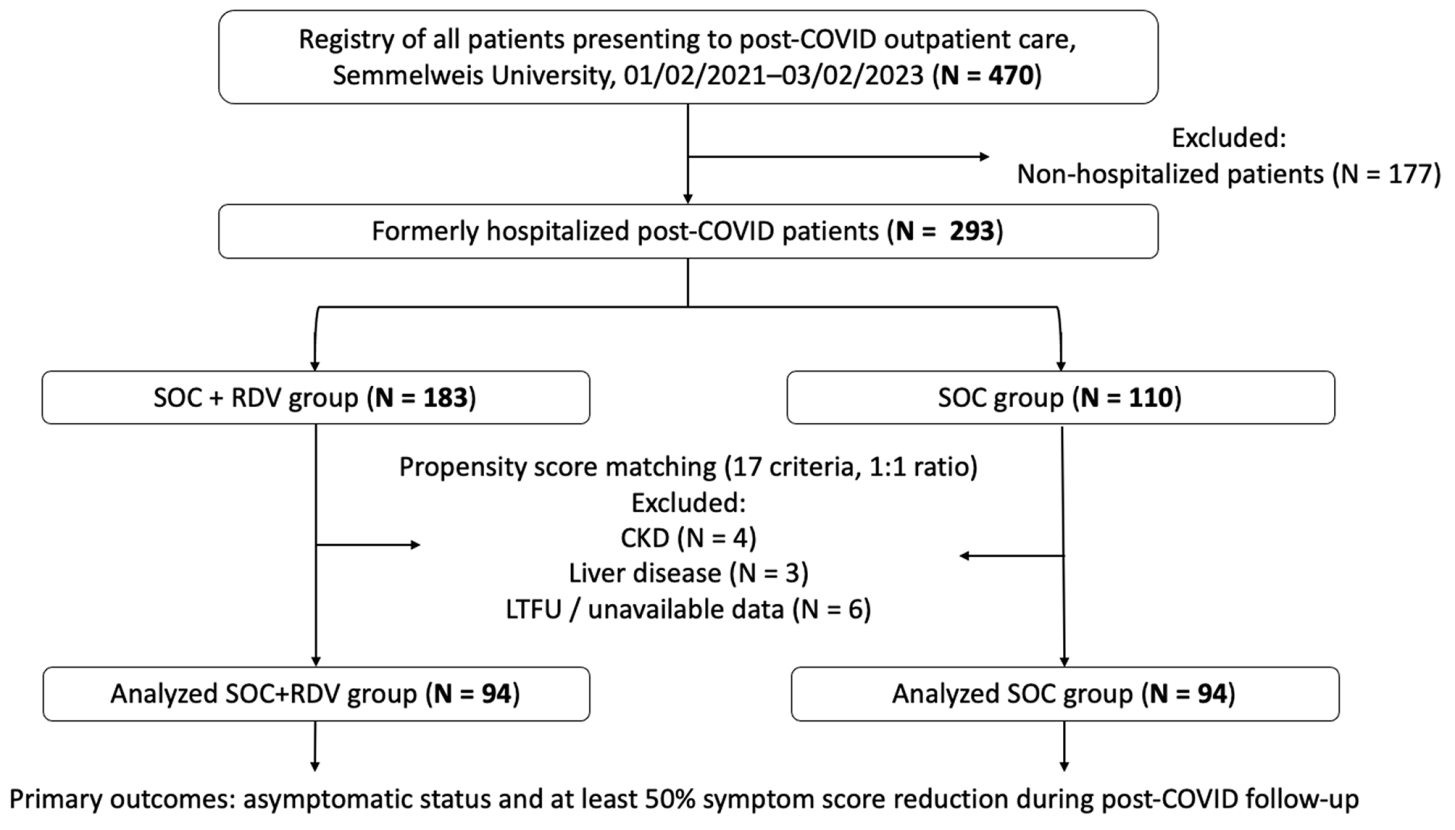
Study flow-chart showing the attainment of the analyzed comparison groups. CKD: chronic kidney disease, LTFU: lost-to-follow-up, RDV: remdesivir, SOC: standard of care

This is a 2-year observational clinical cohort study based on a prospective registry including all consecutive patients presenting to the post-COVID outpatient clinic of the Department of Pulmonology, Semmelweis University, Budapest, Hungary, between 01/02/2021 and 03/02/2023 (*N* = 470). The present study included formerly hospitalized patients (*N* = 293, Supplementary Table 1), divided into two groups by the applied treatment: patients who received antiviral RDV treatment in addition to standard-of-care (SOC), or treated with only SOC. In our statistical analysis, we evaluated the data of two comparable propensity score matched groups based on the applied therapies: SOC + RDV group (SOC + RDV; *N* = 94) vs. only SOC group (SOC; *N* = 94) (Fig. 1). RDV was used in suitable cases where the patient had agreed to the therapy and needed oxygen supplementation according to the actual SmPC, and the drug was available at the treating hospital. Exclusion criteria for this study included chronic kidney disease; liver disease; loss to follow-up; and unavailable or inconsistent data regarding hospitalization, patient history, or symptoms at follow-up. COVID-19 VOCs, counting from the start date of hospitalization, were defined as follows: pre-Delta era before 1st of September 2021; Delta era 1st of September 2021– 1st of January 2022; and Omicron era from 1st of January 2022. Post-COVID care was offered for all symptomatic patients affected by pre-Delta and Delta VOCs, however some asymptomatic patients also applied for evaluation. Furthermore, following Delta VOC, the policy has been changed, and evaluation was offered for all patients.

Outcomes.

We defined asymptomatic status and ≥ 50% symptom score reduction at PCC as the primary outcomes, while quality of life (QoL) parameters, pulmonary function (PF) test, and 6-minute walk test (6MWT) results were the secondary outcomes.

Data collection.

Data on previous health conditions, COVID-19 related history, therapy during hospital stay, symptoms at hospital admission, and during the presence of PCC were collected at post-COVID care visit. Detailed pulmonary function, 6MWT, and QoL questionnaires were assessed [27]. For the measurement of QoL, visual analog scale (VAS) (self-reported QoL on a 0-100 scale), Pittsburg Sleep Quality Index (PSQI) Fatigue Severity Scale (FSS) and Epworth Sleepiness Scale (ESS) as generic health-related quality-of-life instrument were applied [28–31].

Pulmonary function tests assessed forced vital capacity (FVC), forced expiratory volume in 1 s (FEV1), total lung capacity (TLC), and residual volume (RV) in compliance with the guidelines established by the American Thoracic Society and the European Respiratory Society (ATS/ERS) [32]. The diffusing capacity of the lungs for carbon monoxide (DLCO) was evaluated using the single-breath CO method, and the carbon monoxide transfer coefficient (KLCO) was calculated [33]. Respiratory muscle strength was measured by recording maximal inspiratory pressure (PImax) and maximal expiratory pressure (PEmax). All PF measurements were conducted, as in our previous study [11], using the PDD-301/s device (Piston, Budapest, Hungary) [34].

In 72.3% of cases (*N* = 136), chest CT (computed tomography) was performed during hospitalization, and data was collected for lung involvement. Five groups were defined according to lung involvement: <10%, 10–24%, 25–49%, 50–74% and 75%<. When analyzing symptom burden, we established symptom scores based on the number of the symptom domains (fever/chills, cough, dyspnea, fatigue, sleepiness, insomnia, headache, palpitation, smell/taste loss, upper respiratory complaints, gastrointestinal complaints) each patient had during hospitalization and at PCC. These symptom domains were prespecified when the registry was first started, according to institutional protocols for evaluating COVID-19-related symptoms. Thus, they mainly included persisting symptoms of COVID-19 as part of PCC (Questionnaire translated into English in Supplementary File 1).

Data collection, storage, syntactic and semantic validation were performed using a workflow-integrated structured data collection and analysis software provided by Neumann Medical Ltd.

The study was designed in accordance with the 1964 Helsinki Declaration and its later amendments, and the retrospective protocol was approved by the ethical committee of the Semmelweis University Regional and Institutional Committee of Science and Research Ethics (SE RKEB 145/2022 and 147/2022).

### Statistical analysis

To account for potential confounding in this study, we applied propensity score matching (PSM). As the use of ventilation therapy (known predictor for PCC [35]) differed substantially between the two groups, PSM was stratified by ventilation and performed separately in each stratum to ensure the same proportion of ventilated patients in the two groups. Logistic regression was used to estimate the propensity scores, with the following 16 covariates: age, sex, Body-Mass Index (BMI), VOC, chronic obstructive pulmonary disease (COPD), asthma, diabetes, coronary artery disease (CAD), chronic heart failure (CHF), peripheral artery disease (PAD), Stroke/TIA, Charlson Comorbidity Index (the score predicts 10-year survival in patients with several comorbidities) [36], antibiotic therapy, steroids, anticoagulants, oxygen supplementation, favipiravir. Then, a 1:1 nearest-neighbor matching algorithm was run without replacement to establish the comparison groups.

Differences in categorical variables between groups were evaluated by chi-squared test or two-tailed Fisher’s exact test. Continuous variables are presented as mean ± standard deviation (SD) or median and interquartile range (IQR). Student’s T-test or Mann-Whitney U test was applied to compare continuous data based on the distribution of the variables. Kaplan-Meier survival curves were created to visualize, and Cox tests were used to compare the time to asymptomatic status and ≥ 50% symptom score reduction between the groups. Multivariable Cox regression models were applied to assess the effect of other factors and to further eliminate confounding. Covariates were selected based on their association with the variable of interest (RDV), and the pre-defined endpoints. All variables were included in the models that showed a trend for association in univariable regression tests (Supplementary Table 2) and were of clinical relevance. Variables with more than 5% missing values were excluded from the regression models, including lung involvement. For variables with < 5% missing values, simple imputation with median or mode was used. This was performed for BMI (median = 29.6) and VOC (mode = 1, pre-Delta variant). Schoenfeld residuals test was performed to check proportional hazard assumptions. IBM SPSS (IBM Corp., Armonk, NY, USA, version 28) and Stata (StataCorp LLC, College Station, TX, USA, release 18) statistical software packages were used for data analysis. A p-value < 0.05 was defined as statistically significant.

## Results

Patient characteristics, data about COVID-19 hospitalization, and administered therapies for COVID-19 are summarized in Table 1. No differences were noted in age, sex, BMI, underlying comorbidities or Charlson comorbidity scores; and length of hospital stay. Most of the patients were affected by pre-Delta VOCs (SOC: 69.2% vs. SOC + RDV: 73.4%) and there was no significant difference between the two groups regarding VOCs. Most patients who underwent chest-CT (*N* = 136) had at least 10% lung involvement, and severe (≥ 50%) COVID-pneumonia was found in most of the patients (SOC: 55.6% vs. SOC + RDV: 53.5%), without differences between groups. A significant proportion of patients required oxygen, and almost all patients were on antibiotics, steroid and anticoagulant therapy, as standard of care regimen at that time [1]. In the SOC + RDV group, the use of oxygen supplementation (SOC: 80% vs. SOC + RDV: 94%, *p* = 0.005) and steroids (SOC: 88% vs. SOC + RDV: 97%, *p* = 0.027) were higher. There was no significant difference between the 2 groups in the need for non-invasive or invasive ventilation support. Patients receiving only SOC presented for post-COVID pulmonary care later (SOC: median 97 days vs. SOC + RDV: 68 days, *p* = 0.003) as compared to the RDV-treated group. Most patients were still symptomatic (SOC: 66% vs. SOC + RDV: 60%, *p* = 0.449) when presenting at post-COVID pulmonary care.

**Fig. 2:**
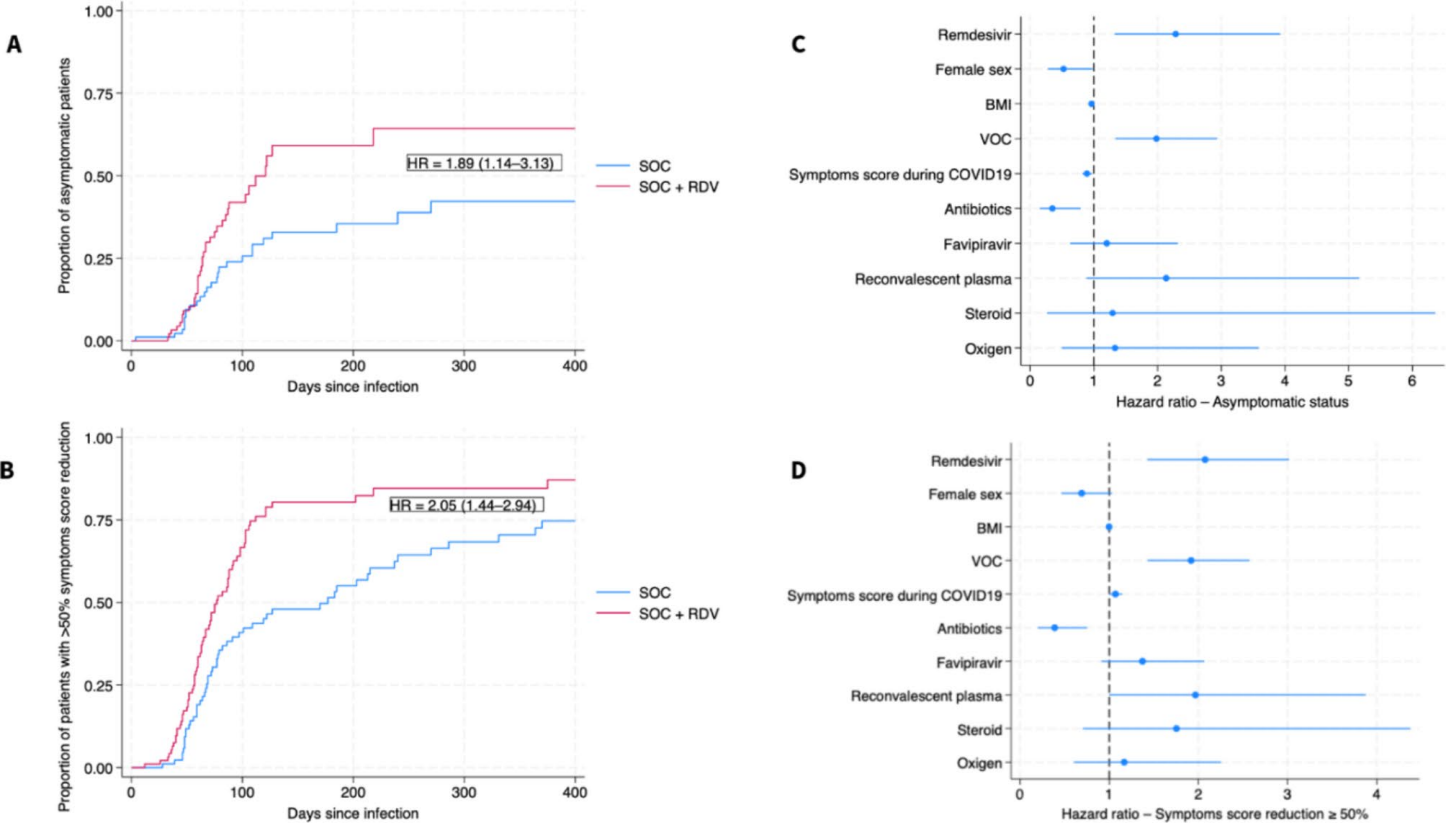
(**A**) Kaplan-Meier curve of the proportion of asymptomatic patients over time since hospital admission (days). (**B**) Kaplan-Meier curve of the proportion of patients ≥ 50% symptom score reduction over time since hospital admission (days). (**C**) Factors influencing asymptomatic status analyzed by Cox regression model with hazard ratios and 95% confidence interval. (**D**) Factors influencing ≥ 50% symptom score reduction analyzed by Cox regression model with hazard ratios and 95% confidence interval. BMI: body mass index, CI: confidence-interval, HR: hazard ratio, RDV: remdesivir, SOC: standard-of-care, VOC: variant of concern

In univariable analysis, asymptomatic status or a minimum 50% reduction of symptom score were reported at an earlier stage after infection in the RDV group compared to the SOC group (unadjusted HR = 1.89, 95% CI = 1.14–3.13, *p* = 0.014; and HR = 2.05, 95% CI = 1.44–2.94, p = < 0.001; respectively) (Fig. 2A-B). Multivariable Cox regression results for the two primary endpoints are displayed in Table 2; Fig. 2C-D. Results show that the use of RDV favors earlier attainment of asymptomatic status (adjusted HR = 2.28, 95% CI = 1.33–3.92, *p* = 0.003) and ≥ 50% symptom burden reduction (adjusted HR = 2.08, 95% CI = 1.43–3.02, *p* < 0.001) after adjustment for other covariates (also including oxygen and steroid use). Later VOC and smaller symptom burden during COVID19 were also associated with faster symptom resolution. The proportional hazards assumption was met for both multivariable models (*p* = 0.320 for (1) model, and *p* = 0.761 for (2) model). Detailed results of univariable Cox regression are displayed in Suppl. Table 2.

Patient-reported symptoms during COVID-19 and PCC period are summarized in detail in Suppl. Tables 3 and Suppl. Figure 1. During COVID-19, the most common symptoms were fatigue, respiratory symptoms (cough, dyspnea), fever and chills, while at PCC many patients still reported fatigue, sleep disturbances, palpitation, and respiratory symptoms (Suppl. Figure 1). Sleep disturbances were less frequent (SOC: 27% vs. SOC + RDV: 14%, *p* = 0.0029) and disappeared in significantly higher proportion in the RDV group after the acute infection (SOC: 30.9% vs. SOC + RDV: 47.9%, *p* = 0.017, Suppl. Table 3).

Regarding QoL parameters, PSQI score results showed significant differences, as RDV treated patient group reported better outcomes (SOC: 7.66 vs. SOC + RDV: 5.90, *p* = 0.025). Although ESS scores showed no differences between the groups, sleep disturbances (sleepiness or insomnia) were common PCC symptoms.

Results of PF tests and 6MWTs are listed in Supplementary Table 4. No significant difference was detected between the groups. Furthermore, FVC and FEV1/FVC were lower than 80% in both groups, suggesting often mild combined ventilatory changes. 6MWT test showed normal values in most cases, desaturation > 3% was detected in 21.5% vs. 28% of the patients. Only heart rate values showed a difference between the two groups, with SOC + RDV patients having higher values.

**Table 1:** Patient characteristics. BMI: body-mass index, CAD: coronary artery disease, CHF: chronic heart failure, COPD: chronic obstructive pulmonary disease, CT: computed tomography, HT: hypertension, PAD: peripheral artery disease, RDV: remdesivir, SD: standard-deviation, SOC: standard-of-care, TIA: transient ischemic attack. Bold P-values indicate significance at 0.05 alpha level

Baseline characteristics of study population	SOC *N* = 94	SOC + RDV *N* = 94	*P*-value
Age, mean ± SD	58 ± 15	60 ± 13	0.293
Sex, N (%)	34 (36.2)	34 (36.2)	1
BMI, mean ± SD	29.2 ± 5.3	30.6 ± 7.6	0.442
HT, N (%)	47 (50.0)	49 (52.1)	0.770
COPD, N (%)	8 (8.5)	8 (8.5)	1
Asthma, N (%)	10 (10.6)	9 (9.6)	0.809
Diabetes, N (%)	6 (6.4)	8 (8.5)	0.578
CAD, N (%)	8 (8.5)	10 (10.6)	0.620
CHF, N (%)	1 (1.1)	1 (1.1)	1
PAD, N (%)	1 (1.1)	2 (2.1)	0.561
Stroke / TIA, N (%)	5 (5.3)	3 (3.2)	0.470
Charlson comorbidity score, mean ± SD	1.87 ± 1.77	1.96 ± 1.56	0.483
**Variants of concern**,** N (%)**	**SOC** *N* = 94	**SOC + RDV** *N* = 94	**P-value**
pre-Delta era	65 (69.2)	69 (73.4)	0.789
Delta era	20 (21.3)	18 (19.2)	
Omicron era	9 (9.6)	7 (7.5)	
**Lung involvment during COVID-19**,** N (%)**	**SOC** *N* = 94	**SOC + RDV** *N* = 94	**P-value**
< 10%	3 (4.8)	1 (1.4)	0.503
10–24%	10 (15.9)	8 (10.1)	
25 − 49%	15 (23.8)	19 (26)	
50–74%	15 (23.8)	19 (26)	
75% <	20 (31.8)	20 (27.4)	
Chest CT missing	31 (33)	21 (22.3)	
**Treatment during COVID-19**,** N (%)**	**SOC** *N* = 94	**SOC + RDV** *N* = 94	**P-value**
Remdesivir	0 (0.0%)	94 (100.0%)	–
Antibiotic	84 (89.4%)	86 (91.5%)	0.620
Favipiravir	29 (30.9%)	21 (22.3%)	0.187
Reconvalescent plasma	4 (4.3%)	9 (9.6%)	0.151
Steroid	83 (88.3%)	91 (96.8%)	**0.026**
Anticoagulant	86 (91.5%)	90 (95.7%)	0.233
Oxygen	75 (79.8%)	88 (93.6%)	**0.005**
Non-invasive ventilation	29 (30.9%)	29 (30.9%)	1
Invasive ventilation	14 (14.9%)	9 (9.6%)	0.266
Length of hospital stay, days (mean ± SD)	18 ± 18	17 ± 14	0.664
**Post-COVID care**	**SOC** *N* = 94	**SOC + RDV** *N* = 94	**P-value**
Days between infection and first visit, median (IQR)	97 [59–215]	68 [56–103]	**0.003**
Symptomatic patients at first visit N (%)	62 (66)	37 (60)	0.449
Asmyptomatic patients at 6 month following infection N (%)	23 (24.5)	36 (38.3)	**0.041**
At least 50% symptom score reduction at 6 month N (%)	40 (42.6)	67 (71.3)	**< 0.001**

**Table 2:** Multivariable Cox regression models. BMI: body mass index, CI: confidence-interval, HR: hazard ratio, IQR: inter-quartile range, RDV: remdesivir, SOC: standard-of-care, VOC: variant of concern

Multivariable Cox regression models	Endpoint of asymptomatic status		Endpoint of > 50% symptom score reduction	
	HR (95% CI)	*P*-value	HR (95% CI)	*P*-value
Remdesivir	2.28 (1.33–3.92)	0.003	2.08 (1.43–3.02)	< 0.001
Female sex	0.52 (0.28–0.98)	0.043	0.69 (0.46–1.03)	0.069
BMI	0.96 (0.92–1.01)	0.139	1.00 (0.97–1.02)	0.881
Later VOC	1.98 (1.34–2.94)	0.001	1.92 (1.43–2.57)	< 0.001
Symptom score during COVID19	0.89 (0.82–0.97)	0.009	1.07 (1.00–1.14)	0.051
Antibiotics	0.35 (0.15–0.79)	0.012	0.39 (0.20–0.75)	0.005
Favipiravir	1.20 (0.62–2.32)	0.581	1.37 (0.91–2.07)	0.127
Reconvalescent plasma	2.13 (0.88–5.17)	0.093	1.97 (1.00–3.88)	0.051
Steroid	3.08 (0.63–15.14)	0.166	1.75 0.70–4.38)	0.229
Oxygen	1.33 (0.49–3.59)	0.571	1.17 (0.61–2.25)	0.644

## Discussion

In our study, we investigated the effect of additional RDV therapy received during COVID-19 hospital stay on PCC outcomes compared to patients only on SOC. In this propensity score matched cohort, the use of RDV was associated with earlier attainment of complete or at least 50% symptom resolution. RDV patients reported less sleep disturbances and better sleep quality. There were no notable differences in functional outcomes including 6MWT and PF tests. Our results indicate a possible beneficial effect of RDV in terms of symptom resolution after COVID19 infection.

According to ACTT-1 and other studies, early RDV treatment may prevent progression to severe disease, reduce mortality and could shorten duration of hospital stay [1, 15, 17–19, 37, 38]. In accordance with these results, our study showed that in our total formerly hospitalized population (*N* = 293) patients receiving RDV treatment spent less time in hospital. Our study is partly based on retrospective data analysis, and the patient cohort was treated across multiple Hungarian hospitals. As a result, certain risk factors for long COVID could not be fully evaluated due to variations in data registration practices across institutions. Specifically, we were unable to calculate a COVID-19 severity score index because some data domains (e.g. oxygen saturation level) were incomplete or missing. However, we were able to obtain data on initial pulmonary CT scans, including lung involvement percentages. To address the severity of COVID-19 in our analysis, we assessed pneumonia severity using these lung involvement metrics as a surrogate indicator. There were no differences between the compared groups in pneumonia severity and ventilation support needs based on the evaluated parameters we had data about. Of all analyzed patients, approximately 60% had severe lung involvement during COVID-19 infection. Severe COVID-19 infection is more often associated with impaired long-term outcomes and higher PCC symptom burden [39, 40].

Prevalence of post-COVID symptoms and PCC are still variable, World Health organization (WHO) defines the prevalence around 10–20% [41]. Associated risk factors for developing PCC are female sex, older age, higher BMI, smoking, comorbidities, previous hospitalization, and intensive care (ICU) admission [40]. In concordance with our study, most frequent patient reported persisting symptoms in previous studies were fatigue, sleep disturbances, anxiety or depression, loss of taste and smell and respiratory symptoms (cough, dyspnea); and symptoms might alter, decrease/increase or disappear by time [9, 42].

An Italian prospective study examined the prevalence and the risk factors of long-covid syndrome (LCS) and evaluated the impact of RDV in developing LCS [26]. They confirmed that severity of illness, ICU admission and length of hospitalization were independent predictors for LCS, while RDV showed a protective effect on the LCS onset; and reduced the post-COVID-19 functional status (PCFS) scale, resulting in better functional status and QoL [26, 43]. Two other studies reported that RDV was associated with less fatigue and cognitive impairment [44] and a significantly shorter duration of PCC was observed when compared with those not receiving RDV [39].

Despite rigorous propensity-score mathcing we acknowledge that Table 1 highlights remaining differences in certain therapies including oxygen supplementation and steroid use. To account for these potential remaning confounding variables, we performed a multivariable regression analysis when assessing the effects of remdesivir. This analysis included oxygen supplementation and steroid use as covariates to adjust for these differences. Our findings indicate that the use of remdesivir was associated with significantly faster patient-reported symptom resolution independent of other analyzed treatments.

Timely administration of another antiviral treatment showed beneficial effect in a large real world data analysis assessing the outcome of nirmatrelvir-ritonavir on PCC: nirmatrelvir-ritonavir was associated with reduced risk of post-covid condition, post-acute death, and post-acute hospitalization as well [45].

Although restrictive ventilatory pattern and CO diffusion reduction after COVID-19 was reported in most of the case series and studies in previous literature [42, 46] our population had mainly physiological PF, with slight reduction in values. Airway involvement over the long term was reported previously, and our data showed slight FEV1/FVC changes as well, but this needs further investigations [47].

Some previous papers have reported the effect of RDV on QoL [25, 26]. A study of 181 patients did not find any difference in QoL after 1 year of hospitalization in patients treated with RDV [25]. Our results show a significant difference in terms of sleep related QoL in PSQI scores; however, our follow-up period was shorter and not standardized. Our QoL questionnaire for sleep disturbances (ESS) has not confirmed any differences between the groups.

Our study has several limitations. Only patients who self-presented to the outpatient post-COVID care were enrolled; therefore, patients with more severe disability were not studied. Groups differed in time elapsed between the infection and first post-COVID visit. Vaccination data is unavailable, as data collection about this was not mandated when the registry began, predating the availability of vaccines. In Hungary, the vaccination campaign initially targeted vulnerable sub-populations (e.g. healthcare workers) and was only later opened for the general population during the late pre-Delta era; therefore, most of the analyzed patients supposedly had not been fully vaccinated by the time of the infection. Recall bias could have occurred for reporting symptoms, most likely biasing results towards the null, thus it could not be the reason for the observed difference. Finally, confounding by indication is possible, as patients were treated in different hospitals, data collection about other potential confounders influencing the use of RDV was not feasible. However, to eliminate measured confounding, rigorous propensity score matching and multivariable analysis were performed using available registry data and data from medical records, while contraindications of RDV were applied as exclusion criteria for the study.

## Conclusion

In this propensity score matched cohort, the use of RDV was associated with earlier attainment of complete or at least 50% symptom resolution during the PCC period, while there were no notable differences in functional outcomes. Our results indicate a possible beneficial effect of RDV in terms of symptom resolution after COVID19 infection.

## Electronic supplementary material

Below is the link to the electronic supplementary material.

Supplementary Material 1

Supplementary Material 2

## Data Availability

The datasets generated during and/or analyzed during the current study are not publicly available but are available from the corresponding author on reasonable request.
