# Supplementary material for: Real-world evidence of remdesivir in formerly hospitalized COVID-19 patients: patient-reported and functional outcomes
Source: BMC Infect Dis. 2025 Jan 9;25:43. doi: 10.1186/s12879-024-10398-w (PMC11715443; doi:10.1186/s12879-024-10398-w)
Supplement: Supplementary file 1 — Supplementary Material 1 [file 12879_2024_10398_MOESM1_ESM.pdf]

Supplementary File 1. Used questionnaire for collecting data on symptoms from local SOP, translated to English. Department of Pulmonology, Semmelweis University, Post-COVID Standard Operating Procedures version 03.02.2021

Symptom	During acute COVID19 infection	After hospital discharge	Currently
Fever, chills			
Dyspnea			
Fatigue			
Muscle ache			
Sleepiness			
Insomnia			
Headache			
Palpitation			
Loss of taste or smell			
Sore-throat			
Rhinorrhea			
Nausea, vomiting			
Diarrhea			
Other			
