# Supplementary material for: Real-world evidence of remdesivir in formerly hospitalized COVID-19 patients: patient-reported and functional outcomes
Source: BMC Infect Dis. 2025 Jan 9;25:43. doi: 10.1186/s12879-024-10398-w (PMC11715443; doi:10.1186/s12879-024-10398-w)

**Supplementary Table 1.** Patient characteristics of the total formerly hospitalized cohort.

|  | **ALL (N=293)** | **SOC+RDV treatment (N=183)** | **SOC treatment**  **(N=110)** | **p** |
| --- | --- | --- | --- | --- |
| **Age (years)** | 57.9±14.1 | 57.4±14.2 | 58.6±14.1 | 0.500 |
| **Sex N (%)** |  | | | |
| **female** | 109 (37.2) | 66 (36.1) | 43 (39.1) | 0.603 |
| **male** | 184 (62.8) | 117 (63.9) | 67 (60.9) |  |
| **BMI (kg/m^2^)** | 30.8±6.7 | 31.5±7.1 | 29.6±5.8 | **0.018** |
| **Days of hospitalization** | 16±16 | 14±13 | 19±19 | **0.022** |
| **Distribution by variants of concern N (%)** | | | | |
| pre-Delta era | 168 (57.3) | 102 (55.7) | 66 (60.0) | 0.076 |
| Delta era | 82 (28.0) | 56 (30.6) | 26 (23.6) |  |
| Omicron era | 37 (12.6) | 24 (13.1) | 13 (11.8) |  |
| Missing data | 6 (2.1) | 1 (0.6) | 5 (4.6) | - |
| **Days between emission and post-COVID care admission** | 105±127 | 81±94 | 146±161 | **<0.005** |
| **Additional therapy during COVID-19 N (%)** | | | | |
| Antibiotics | 265 (90.0) | 168 (91.8) | 97 (88.2) | 0.310 |
| Favipiravir | 51 (17.4) | 21 (11.5) | 30 (27.3) | **<0.005** |
| Convalescent plasma | 19 (6.5) | 15 (8.2) | 4 (3.6) | 0.120 |
| Steroids | 278 (94.9) | 180 (98.4) | 98 (89.1) | **<0.005** |
| Anticoagulants | 278 (94.9) | 176 (96.2) | 102 (92.7) | 0.190 |
| Oxygen | 268 (91.5) | 177 (96.7) | 91 (82.7) | **<0.005** |
| Non-invasive ventilation | 75 (25.6) | 31 (16.9) | 44 (40.0) | **<0.005** |
| Invasive ventilation | 27 (9.2) | 9 (4.9) | 18 (16.4) | **0.001** |
| **Symptoms during and after COVID-19** | indicated in Tabel 2 and Table 3 | | | |
| **Post-COVID symptomatic patients N (%)** | 183 (62.5) | 112 (61.2) | 71 (64.5) | 0.567 |
| **Lung involvement during COVID-19 N (%)** | | | | |
| No lung involvement/ <5% | 5 (1.7) | 2 (1.1) | 3 (2.7) | 0.320 |
| Lung involvement 5-49% | 89 (30.4) | 63 (34.4) | 26 (23.6) |  |
| Lung involvement ≥50% | 135 (46.1) | 89 (48.6) | 46 (41.8) |  |
| Chest-CT missing | 64 (21.8) | 29 (15.9) | 35 (31.9) | - |
| **Lung function test at post-COVID care** | | | | |
| FVC (L) | 3.2±0.97 | 3.3±0.99 | 3.2±0.92 | 0.330 |
| FVC (%) | 79.5±15.5 | 80.0±15.4 | 78.8±15.8 | 0.570 |
| FEV_1_ (L) | 2.7±0.9 | 2.7±0.86 | 2.7±0.83 | 0.301 |
| FEV_1_ (%) | 84.7±17.6 | 85.2±16.3 | 83.8±19.6 | 0.293 |
| FEV_1_/FVC (%) | 75.7±25.6 | 76.8±24.4 | 74.0±24.5 | 0.374 |
| TLC (L) | 5.4±1.5 | 5.3±1.5 | 5.5±1.4 | 0.578 |
| TLC (%) | 92.0±24.2 | 90.5±22.7 | 94.3±26.5 | 0.227 |
| RV (L) | 2.1±1.0 | 2.0±0.95 | 2.2±1.1 | 0.944 |
| RV (%) | 99.0±44.8 | 97.6±42.2 | 101.4±49.0 | 0.556 |
| DL_CO_ (mmol/min/kPa) | 8.6±2.7 | 8.6±2.8 | 8.6±2.7 | 0.773 |
| DL_CO_ (%) | 100.2±25.0 | 100.6±24.3 | 99.4±26.1 | 0.694 |
| Klco (mmol/min/kPa) | 1.6±0.4 | 2.4±0.4 | 1.6±0.4 | 0.299 |
| KL_CO_ (%) | 104.5±26.9 | 104.3±25.2 | 105.0±29.5 | 0.874 |
| PImax | 8.0±3.3 | 8.2±3.4 | 7.7±3.3 | 0.304 |
| PEmax | 9.3±3.3 | 9.5±3.3 | 9.0±3.1 | 0.320 |
| **6MWT at post-COVID care** | | | | |
| Distance (m) | 435±123 | 440±118 | 426±133 | 0.403 |
| Heart rate at start (1/min) | 85.5±13.9 | 87.4±13.4 | 82.2±14.2 | **<0.005** |
| Heart rate at end (1/min) | 116.6±21.6 | 119.2±21.1 | 112.2±21.8 | **0.014** |
| Saturation at start (%) | 96.6±1.7 | 96.4±1.6 | 96.8±2.0 | 0.373 |
| Saturation at end (%) | 91.6±6.0 | 91.5±5.0 | 91.7±7.2 | 0.940 |
| Patients with desaturation* N (%) | 70 (23.9) | 43 (23.5) | 27 (24.5) | 0.839 |
| BORG at start (0-10) (median) [range] | 0 [0-60] | 0 [0-60] | 0 [0-30] | 0.682 |
| BORG at end (0-10) (median) [range] | 10 [0-80] | 10 [0-80] | 10 [0-70] | 0.531 |
| **Quality of life at post-COVID care** | | | | |
| Visual analog scale | 73.3±17.7 | 75.2±15.9 | 70.3±19.9 | **0.030** |
| ESS score (median) [range] | 6 [0-27] | 6 [0-27] | 6 [0-14] | 0.71 |

*>3% desaturation; 6MWT: 6-minute walk test; BMI: body mass index; DLCO: transfer factor for carbon monoxide; ESS: Epworth Sleepiness Scale; FEV1: forced expiratory volume in 1^st^ second of exhalation; FVC: forced vital capacity; KLCO: transfer coefficient for carbon monoxide; PEmax: maximal expiratory mouth pressure; PImax: maximal inspiratory mouth pressure; RDV: remdesivir; RV: residual volume; SOC: standard of care; TLC: total lung capacity.

**Supplementary Table 2.** Univariable Cox regression results.

| **Univariable Cox regression models** | **Endpoint of asymptomatic status** | | **Endpoint of >50% symptom score reduction** | |
| --- | --- | --- | --- | --- |
|  | HR  (95% CI) | P-value | HR  (95% CI) | P-value |
| Age | 0.99  (0.98 - 1.02) | 0.944 | 1.00  (0.99–1.01) | 0.930 |
| Female sex | 0.4  (0.22 - 0.72) | 0.002 | 0.61  (0.42–0.89) | 0.011 |
| BMI | 0.96  (0.92 - 1.004) | 0.072 | 1.00  (0.98–1.03) | 0.960 |
| Charlson-score | 0.95  (0.82 - 1.11) | 0.522 | 0.96  (0.86–1.07) | 0.428 |
| VOC | 2.18  (1.54 - 3.07) | <0.001 | 1.77  (1.36–2.32) | <0.001 |
| Symptom-score during COVID-19 | 0.86  (0.80 - 0.94) | <0.001 | 1.05  (0.99–1.12) | 0.102 |
| Remdesivir | 1.89  (1.14 - 3.13) | 0.014 | 2.05  (1.44–2.94) | <0.001 |
| Antibiotics | 0.43  (0.20 - 0.91) | 0.028 | 0.54  (0.30–0.99) | 0.046 |
| Favipiravir | 0.647  (0.36 - 1.17) | 0.15 | 1.03  (0.70–1.50) | 0.893 |
| Reconvalescent plasma | 1.94  (0.83 - 4.54) | 0.127 | 1.74  (0.91–3.35) | 0.096 |
| Steroid | 3.06  (0.74 - 12.57) | 0.122 | 1.79  (0.83–3.85) | 0.139 |
| Anticoagulant | 2.06  (0.50 - 8.47) | 0.317 | 1.10  (0.51–2.37) | 0.800 |
| Oxygen | 1.99  (0.85 - 4.69) | 0.115 | 1.80  (1.04–3.14) | 0.037 |
| Non-invasive ventilation | 1.36  (0.82 - 2.26) | 0.235 | 1.07  (0.73–1.56) | 0.734 |
| Invasive ventilation | 0.92  (0.47 - 1.82) | 0.817 | 0.63  (0.36–1.09) | 0.100 |

**Supplementary Table 3:** Patient reported symptoms during COVID-19 and PCC period; resolution of symptoms experienced during COVID-19 infection.

| **Symptoms during**  **COVID-19, N (%)** | **SOC**  **N = 94** | **SOC + RDV**  **N = 94** | **P-value** |
| --- | --- | --- | --- |
| Fever, chills | 67 (71.3%) | 74 (78.7%) | 0.238 |
| Cough | 66 (70.2%) | 71 (75.5%) | 0.412 |
| Dyspnea | 74 (78.7%) | 69 (73.4%) | 0.393 |
| Fatigue | 70 (74.5%) | 76 (80.9%) | 0.293 |
| Sleep disturbance (sleepiness and /or insomnia) | 35 (37.2%) | 42 (44.7%) | 0.299 |
| Headache | 28 (29.8%) | 34 (36.2%) | 0.352 |
| Palpitation | 36 (38.3%) | 40 (42.6%) | 0.552 |
| Smell and taste loss | 36 (38.3%) | 30 (31.9%) | 0.359 |
| Upper respiratory | 41 (43.6%) | 44 (46.8%) | 0.66 |
| Gastrointestinal | 35 (37.2%) | 37 (39.4%) | 0.764 |
| **PCC symptoms, N (%)** | **SOC**  **N = 94** | **SOC + RDV**  **N = 94** | **P-value** |
| Fever, chills | 3 (3.2%) | 3 (3.2%) | 1 |
| Cough | 25 (26.6%) | 18 (19.1%) | 0.224 |
| Dyspnea | 26 (27.7%) | 24 (25.5%) | 0.741 |
| Fatigue | 43 (45.7%) | 43 (45.7%) | 1 |
| Sleep disturbance (sleepiness and /or insomnia) | 25 (26.6%) | 13 (13.8%) | **0.029** |
| Headache | 11 (11.7%) | 4 (4.3%) | 0.060 |
| Palpitation | 18 (19.1%) | 16 (17.0%) | 0.705 |
| Smell and taste loss | 7 (7.4%) | 3 (3.2%) | 0.194 |
| Upper respiratory | 15 (16.0%) | 9 (9.6%) | 0.190 |
| Gastrointestinal | 5 (5.3%) | 5 (5.3%) | 1 |
| **Resolution of symptoms, N (%)** | **SOC**  **N = 94** | **SOC + RDV**  **N = 94** | **P-value** |
| Fever, chills | 64 (68.1%) | 71 (75.5%) | 0.257 |
| Cough | 44 (46.8%) | 54 (57.4%) | 0.144 |
| Dyspnea | 49 (52.1%) | 47 (50.0%) | 0.770 |
| Fatigue | 32 (34.0%) | 37 (39.4%) | 0.449 |
| Sleep disturbance (sleepiness and /or insomnia) | 29 (30.9%) | 45 (47.9%) | **0.017** |
| Headache | 21 (22.3%) | 30 (31.9%) | 0.140 |
| Palpitation | 23 (24.5%) | 28 (29.8%) | 0.412 |
| Smell and taste loss | 29 (30.9%) | 27 (28.7%) | 0.750 |
| Upper respiratory | 30 (31.9%) | 36 (38.3%) | 0.359 |
| Gastrointestinal | 32 (34) | 32 (34) | 1 |

**Supplementary Table 4.:** Pulmonary function test and 6MWT results.

| **Lung function tests at first visit** | **SOC**  **N = 94** | **SOC + RDV**  **N = 94** | **P-value** |
| --- | --- | --- | --- |
| FVC, L | 3.26 ± 0.89 | 3.17 ± 1.02 | 0.181 |
| FVC, % | 79.59 ± 15.92 | 78.98 ± 16.97 | 0.647 |
| FEV1, L | 2.73 ± 0.81 | 2.68 ± 0.88 | 0.278 |
| FEV1, % | 84.52 ± 19.44 | 85.25 ± 18.72 | 0.998 |
| FEV1 / FVC, % | 73.38 ± 28.06 | 77.08 ± 24.17 | 0.339 |
| TLC, L | 5.56 ± 1.45 | 5.31 ± 1.45 | 0.209 |
| TLC, % | 95.65 ± 27.92 | 91.40 ± 24.95 | 0.666 |
| RV, L | 2.19 ± 1.13 | 2.14 ± 0.97 | 0.871 |
| RV, % | 101.33 ± 52.05 | 102.35 ± 43.69 | 0.534 |
| FEF25 | 92.94 ± 28.38 | 97.83 ± 22.03 | 0.493 |
| FEF50 | 79.30 ± 30.87 | 85.00 ± 27.40 | 0.219 |
| FEF75Lsec | 1.09 ± 0.59 | 1.14 ± 0.63 | 0.879 |
| FEF75 | 122.53 ± 68.07 | 136.63 ± 73.81 | 0.149 |
| FEF2575Lsec | 3.68 ± 4.28 | 3.38 ± 1.31 | 0.775 |
| FEF2575 | 115.38 ± 43.59 | 122.85 ± 37.97 | 0.153 |
| DL_CO_, mmol/min/kPa | 8.82 ± 2.62 | 8.39 ± 2.80 | 0.099 |
| DL_CO_, % | 100.70 ± 25.48 | 98.98 ± 23.84 | 0.526 |
| KL_CO_, mmol/min/kPa | 1.60 ± 0.40 | 1.53 ± 0.38 | 0.144 |
| KL_CO_, % | 106.32 ± 28.44 | 101.14 ± 24.83 | 0.115 |
| PImax, kPa | 7.81 ± 3.30 | 7.98 ± 3.39 | 0.857 |
| PEmax, kPa | 9.10 ± 3.13 | 9.30 ± 3.32 | 0.897 |
| **6MWT at first visit** | **SOC**  **N = 94** | **SOC + RDV**  **N = 94** | **P-value** |
| Distance, m | 436.25 ± 130.71 | 429.35 ± 106.24 | 0.242 |
| Saturation at start (%) | 97.05 ± 1.54 | 96.50 ± 1.30 | 0.066 |
| Saturation at end (%) | 92.24 ± 7.21 | 91.42 ± 4.73 | 0.088 |
| Patients with >3% desaturation, N (%) | 20 (21.5) | 26 (28.0) | 0.308 |
| Heart rate at start (1/min) | 80.76 ± 14.17 | 86.73 ± 14.28 | 0.008 |
| Heart rate at end (1/min) | 111.65 ± 21.90 | 117.54 ± 21.15 | 0.015 |
| BORG at start (0-10) (median) [range] | 3.47 ± 8.35 | 3.10 ± 8.78 | 0.379 |
| BORG at end (0-10) (median) [range] | 15.06 ± 17.26 | 14.35 ± 15.58 | 0.938 |
| **Quality of life at first visit** | **SOC**  **N = 94** | **SOC + RDV**  **N = 94** | **P-value** |
| Visual analog scale | 70.51 ± 20.26 | 74.90 ± 15.16 | 0.108 |
| PSQI score | 7.66 ± 5.27 | 5.90 ± 4.12 | 0.025 |
| FSS score | 39.47 ± 17.92 | 35.69 ± 17.84 | 0.161 |
| ESS score | 6.53 ± 4.41 | 6.35 ± 4.53 | 0.794 |

**Supplementary Figure 1.** Symptoms during COVID-19 infection and at the first post-COVID visit. PCC: post-acute sequalae COVID19**,** RDV: remdesivir, SOC: standard of care.


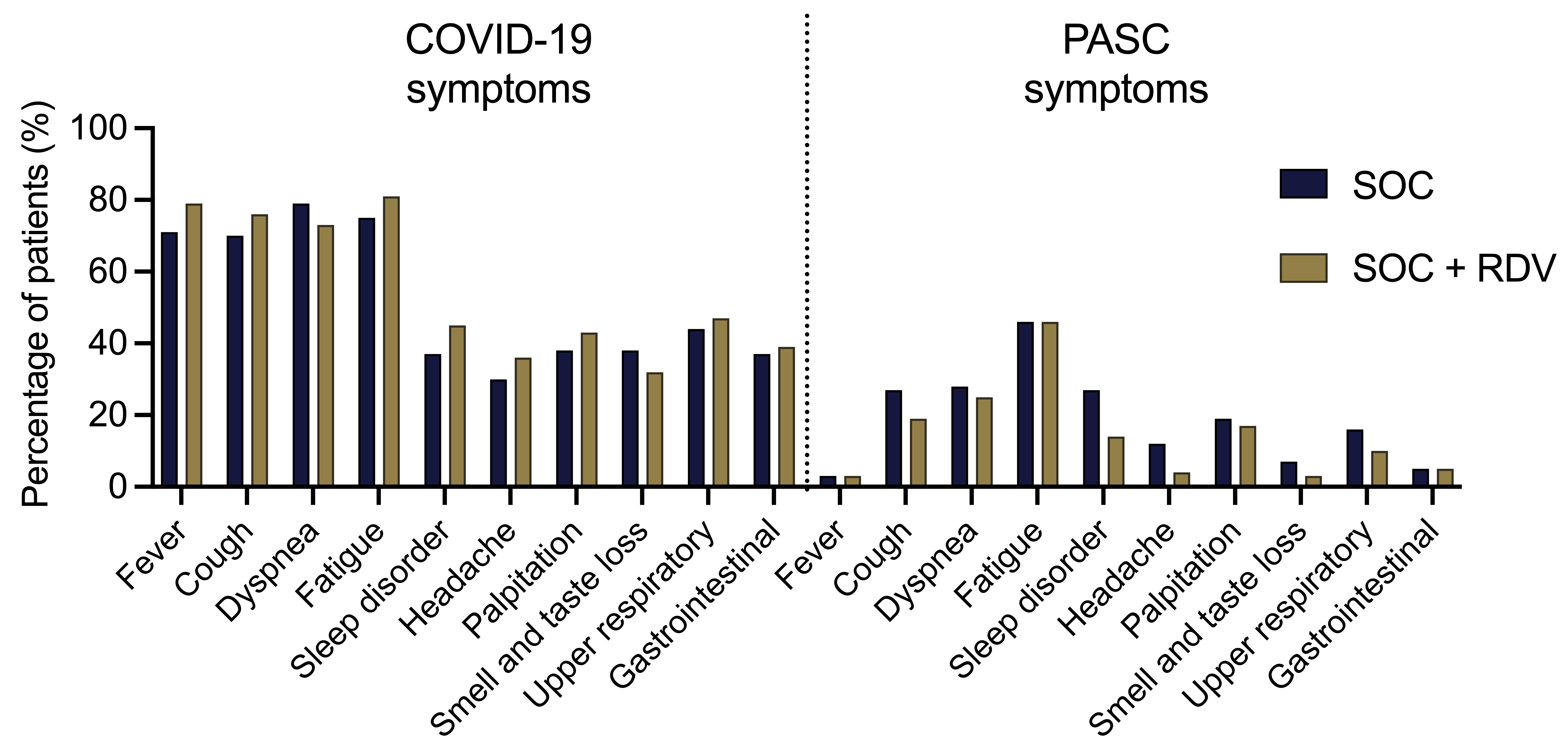

Supplement: Supplementary file 2 — Supplementary Material 2 [file 12879_2024_10398_MOESM2_ESM.docx]
